# Th17 and CD24^hi^CD27^+^ regulatory B lymphocytes are biomarkers of response to biologics in rheumatoid arthritis

**DOI:** 10.1186/s13075-017-1244-x

**Published:** 2017-02-10

**Authors:** Sarah Salomon, Caroline Guignant, Pierre Morel, Gauthier Flahaut, Clément Brault, Clément Gourguechon, Patrice Fardellone, Jean-Pierre Marolleau, Brigitte Gubler, Vincent Goëb

**Affiliations:** 1 Rheumatology Department & EA 4666, Amiens University Hospital, University of Picardie-Jules Verne, Amiens, France; 2 Immunology laboratory & EA 4666, Amiens University Hospital, University of Picardie-Jules Verne, Amiens, France; 3 Hematology laboratory & EA 4666, Amiens University Hospital, University of Picardie-Jules Verne, Amiens, France

**Keywords:** Rheumatoid arthritis, Biomarker, Lymphocyte, Th17, Breg, Treg, Response, Remission, Abatacept, Anti-TNF

## Abstract

**Background:**

The aim was to describe the regulatory B and T cells (Breg and Treg) and T helper 17 (Th17) lymphocytes before and under treatment with biologic drugs, and to assess their potential predictive value as biomarkers of response in rheumatoid arthritis (RA).

**Methods:**

This was a non-randomised, single-centre, prospective study. Patients with active RA (American College of Rheumatology (ACR)/European League Against Rheumatism (EULAR) 2010) who required the initiation or switch to any biologic drug except rituximab were included. The main judgement criterion was the frequency and absolute number of CD24^hi^CD27^+^ Breg and CD24^hi^CD38^hi^ T2/Breg cells, CD25^hi^CD127^low^ Treg and CD45RA^−^CD161^+^CCR6^+^ Th17 cells measured at inclusion in both patients and controls, and after 1, 3 and 6 months of treatment (M1, M3 and M6) in patients with RA, and compared with the M6 response to treatment (EULAR response and Disease Activity Score in 28 joints (DAS28) remission).

**Results:**

Thirty-one patients with RA and 17 controls were included. There was a reduction in T2/Breg frequency at M0 in patients (*p* < 0.001) and absolute numbers (*p* = 0.014) and in immunopositive vs*.* immunonegative RA (*p* = 0.016). DAS28 remission at M6 was associated with increased frequency of Treg (*p* = 0.01). A higher level of CD24^hi^CD27^+^ Breg at baseline was associated with DAS28 remission at M6 (*p* = 0.04) and a good EULAR response at M6 for abatacept-treated patients (*p* = 0.01). A lower M0 level of Th17 was associated with a good EULAR response at M6 (*p* = 0.007), notably under anti-cytokine drugs (*p* = 0.048).

**Conclusions:**

Altogether, these data, although preliminary, suggest that phenotyping of T and B cells has potential value for the stratification of biologic drugs, notably with respect to choosing between abatacept and anti-cytokine blockade.

## Background

The development of different targeted immunotherapies and treatment strategies (treat to target (T2T) and tight control) has allowed significant improvement in the prognosis of rheumatoid arthritis (RA), and long-lasting clinical remission may now be a consideration [1]. Approximately one third of patients, however, experience primary non-response or insufficient response to these different drugs [2, 3]. The search for biomarkers that could predict response to biologic drugs is therefore crucial, in order to ensure that the treatment chosen will be sufficiently effective to halt the progress of the disease, whilst avoiding prescribing patients ineffective, potentially deleterious (leading to infectious or neoplastic complications, etc.) and expensive treatment [4].

Different lymphocyte subpopulations play a major role in the physiopathology of RA, such as regulatory T cells (Treg), T helper 17 (Th17) cells and regulatory B lymphocytes (Breg). In the physiological condition, CD4 + CD25^hi^FoxP3^+^ regulatory T cells constitute 5–10% of the population of CD4^+^ T lymphocytes in the blood [5]. A functional deficit in Treg with a reduction of their suppressive activity has been observed in RA [6]. A loss of Treg capacity to suppress effector CD4^+^CD25^−^ T cells has been observed, prior to anti-TNF treatment for active RA, and a recovery of this defect was observed in patients who respond clinically to anti-TNF drugs with a quantitative increase in Treg frequencies post treatment [6]. Moreover, the quantitative restoration of the Treg upon achieving remission with anti-TNF has been associated with greater probability of long-term remission [7]. An increase in T lymphocytes, and Treg in particular, was furthermore recorded after the use of abatacept, a biologic drug that reproduces the physiological inhibitor role of CTLA4 [8].

The identification of the Th17 lymphocytes, a subpopulation of CD4^+^ T-lymphocytes that produces the highly proinflammatory cytokine IL-17 [9, 10], opened the way for new understanding of the physiopathological and new treatment options (secukinumab or ixekizumab) for RA [11, 12]. Th17 cells are characterised by considerable plasticity (interconversion with Th1 cells), which themselves can also develop into Th17 under some pathophysiological circumstances [13–16]. An inverse correlation between blood Th17 and anti-citrullinated peptide antibodies (ACPA) levels has been observed in patients with RA [17], illustrating the interrelationship between this subpopulation and humoral immunity in this autoimmune rheumatism. The role of Th17 appears to be particularly relevant during the onset of RA and later may be influenced by treatments, increasing under methotrexate (MTX) [18] and decreasing in patients that do not respond to infliximab [19].

B lymphocytes and humoral immunity also play a key role in RA, notably with ACPA and rheumatoid factor (RF) production [20]. Breg, by analogy to Treg, represent a subpopulation of B cells that have immune-regulating properties [21] and play a role in peripheral tolerance [22], mainly via the substantial secretion of IL-10 [22, 23]. Discrepancies in the phenotype of human Breg have been described [24–28], although the most commonly accepted is the inclusion of putative Breg within the immature transitional T2 population (CD19+CD24^hi^CD38^hi^). Additional regulatory properties have been described in the memory B cells CD24^hi^CD27^+^ phenotype (also producing IL-10) [29]. These cells are reported to be able to inhibit the proliferation of CD4^+^ T cells differentiating between Th1 and Th17 cells, producing different proinflammatory cytokines. Although they are reported to be quantitatively and functionally altered in patients with active RA (reduction of their capacity to induce both Treg from CD4+ and prevent the conversion of Treg into Th17 cells) [30, 31], a recent report observed that a high baseline level of Breg appeared to be predictive of a clinical European League Against Rheumatism (EULAR) response at 3 months upon anti-TNF treatment [25].

Thus, the main aim of our study was to describe the different lymphocyte subpopulation frequencies in RA (regulatory B and T cells and Th17 lymphocytes) before and under biologic drugs, in order to test the correlation with subsequent response to the drug received and to identify potential biomarker value that could be used to predict therapeutic response.

## Methods

### Patients and controls

Patients who met the American College of Rheumatology (ACR)/EULAR 2010 criteria for RA [32] with active disease despite receiving a conventional synthetic or biologic disease-modifying antirheumatic drug (DMARD) and who required the initiation or a switch to any biologic drug except rituximab, in association with MTX, were prospectively included in this study. The exclusion criteria were the use of corticosteroid therapy, concurrent or within the last 3 months, at a dose of more than 10 mg/day or treatment with other immunosuppressive therapy than MTX. At least five half-lives were required before changing the treatment if a biologic drug was previously used. Adult patients with a confirmed “mechanical” pathologic condition (osteoporosis, osteoarthritis, etc.) who did not have a personal medical history of neoplastic, inflammatory or infectious diseases were included as controls. All patients and controls signed a consent form to participate in this study, approved by the medical ethics committee of Amiens University hospital, France (number 2015-000833-64).

Different parameters were gathered at inclusion (month 0 (M0)): history and treatments, body mass index (BMI), disease activity evaluated by calculation of the Disease Activity Score in 28 joints (DAS28) and the Health Assessment Questionnaire (HAQ), the erosive nature of the RA (evaluated on radiographs of both hands and feet), the autoimmune status (RF and ACPA assessed by anti-cyclic citrullinated peptide (anti-CCP2 tests)), complete blood count (CBC), measurement of C-reactive protein (CRP) and erythrocyte sedimentation rate (ESR). Patients underwent the same clinical and biological monitoring at 1 (M1), 3 (M3) and 6 months (M6) following treatment.

### Analysis of lymphocyte subpopulations by flow cytometry

Flow cytometry (CMF) was used to conduct lymphocyte phenotyping of fresh blood (Navios A80706, 3 lasers, 10 colours) at M0 in patients and controls and then at M1, M3 and M6 in patients only. We used standard protocols developed under routine immunology hospital services with a view to be able to transfer data into clinical practice with limited additional work. The following monoclonal antibodies conjugated with a fluorochrome were used for the B cell phenotyping: CD19-AA750 (APC-AlexaFluor750), CD24-PC5.5 (phycoerythrin-cyanine) and CD27-PC7, CD38-APC (allophycocyanin). The following antibodies were used for T cell phenotyping: CD3-AA750, CD4-PB, CD45RA-FITC, CD62L-ECD, CD25-PC5.5, CD161-PC7, CD127-AA700 and CCR6(CD196)-APC. All the antibodies came from Beckmann-Coulter® (except CCR6-APC from BioLegend®). At least 20,000 cells were analysed for each sample.

**Fig. 1:**
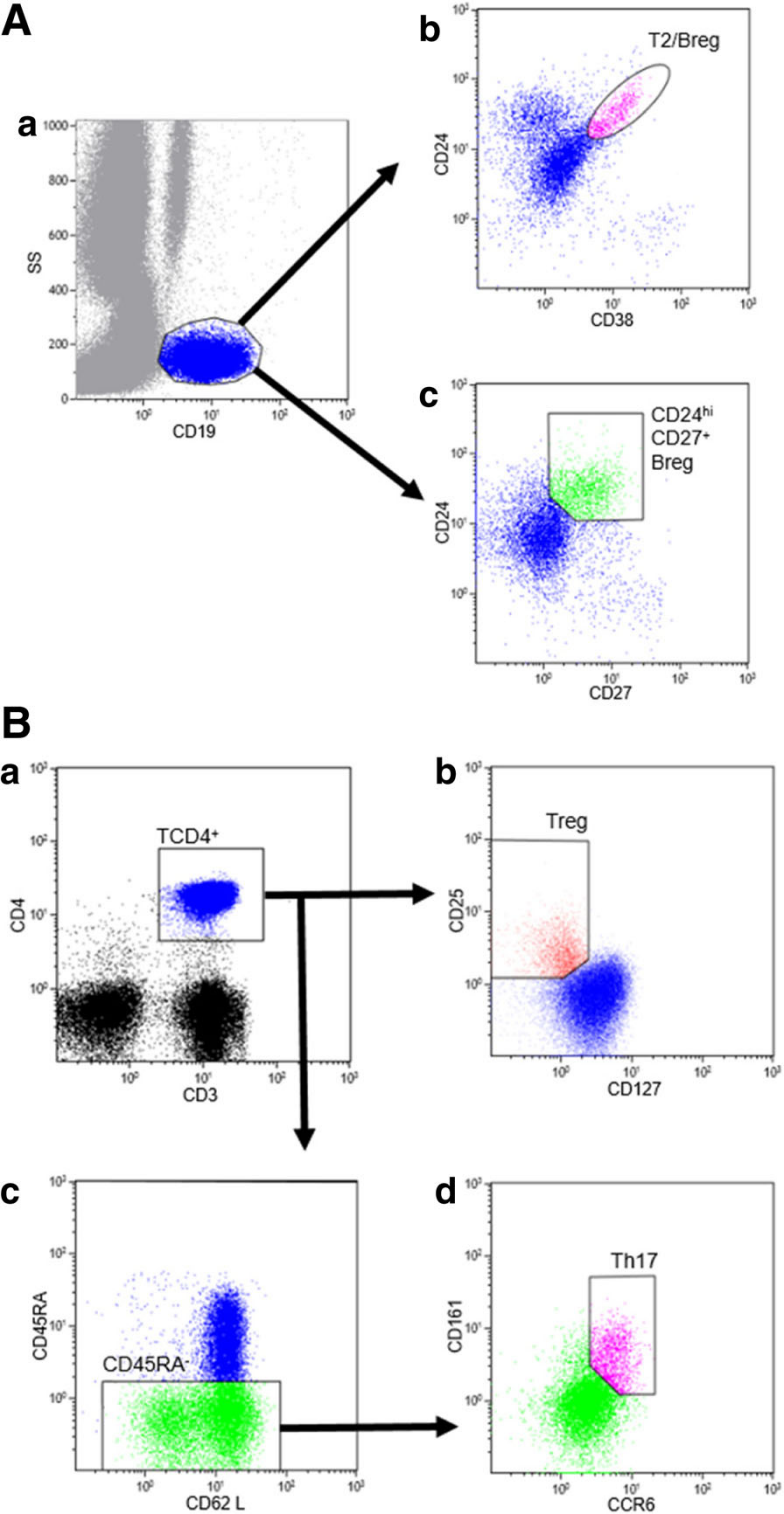
Analysis of lymphocyte subpopulations by flow cytometry: gating strategy. **A** Gating strategy for B lymphocytes; **a** expressed as a single parameter of CD19 and scatter; **b** T2/Breg (CD24^hi^CD38^hi^); **c** CD24^hi^CD27^+^ Breg. **B** Gating strategy for T lymphocytes; **a** CD3^+^CD4^+^; **b** Treg (CD25^hi^CD127^low^); **c** CD4^+^45RA^−^; **d** Th17 (CD45RA^−^CD161^+^CCR6^+^). *Breg* B regulatory cells, *Treg* T regulatory cells, *Th17* T helper 17

The T and B cell populations were identified on the single-parameter expression of CD3 and CD19, respectively, combined with scatter (gating strategies are described in Fig. 1). Regulatory B cells were defined as CD19^+^CD24^hi^CD38^hi^ further referred to as T2/Breg. We also assessed the CD19^+^CD24^hi^CD27^+^ subpopulations. Treg were identified as the CD4 + CD25^hi^CD127^low^ subpopulation as previously described [33]. Th17 cells were identified as subpopulation CD45RA+CD161^+^CCR6^+^ within the CD3 + CD4^+^ T cell gate. The results were expressed as percentage of the parental lineage gate and absolute value per millimeter cubed.

### Statistical analysis

The characteristics of patients and controls were recorded as a percentage for the category-based variables and as the median and 25^th^ and 75^th^ percentiles (interquartile range (IQR)) for continuous variables. The differences in percentage or absolute value of the lymphocyte subpopulations were evaluated using the parametric *t* test, the non-parametric Mann–Whitney or Wilcoxon tests for matched data, or the Friedman test for multiple comparisons. Correlation between the different lymphocyte subpopulations was evaluated using the Spearman test. The general linear model (GLM) and MIXED procedures were used for the repetitive analysis models. We used *p* < 0.05 as the significance threshold for the statistical tests, corrected by the number of tests carried out, where necessary. The statistical analyses were carried out using the Statistical Package for Social Sciences (SPSS) and Statistical Analysis System (SAS) 9.3 software.

## Results

### Baseline characteristics of patients and controls

Table 1 gives the demographic and clinical characteristics of the 31 patients and 17 controls, included prospectively between November 2014 and October 2015. No statistically significant difference was observed. Two patients left the study at M1 and three left at M6.

**Table 1:** Baseline characteristics of patients and controls

		Patients with RA (n = 31)	Control group (n = 17)	*p*
Women		58.1% (n = 18)	41.2% (n = 7)	0.367
Age		57 (48–61)^a^	41 (29–68)^a^	0.131
BMI (kg/m2)		25.8 (22.2–33)^a^	24.0 (22.16–26.30)^a^	0.343
Medical history of smoking		48.4% (n = 15)	23.5% (n = 4)	0.127
Consumption (packets per year)		20 (11–28)^a^	0 (0–5)^a^	0.094
Recent RA ≤2 years		32.3% (n = 10)	-	
Antibodies				
RF+		74.2% (n = 23)	-	
ACPA+		80.6% (n = 25)	-	
Structural damage (erosions)		41.9% (n = 13)	-	
NSAID		12.9% (n = 4)	-	
Corticoids		54.8% (n = 17)	-	
Median dosage (mg/day)		5 (5–6)^a^	-	
Methotrexate		64.5% (n = 20)	-	
Median dose (mg/week)		15 (10–20)^a^	-	
Initiation (1st line of biomedicine)		54.8% (n = 17)	-	
Abatacept		38.7% (n = 12)	-	
Tocilizumab		19.4% (n = 6)	-	
Anti-TNF drugs	Golimumab	16.1% (n = 5)	-	
	Etanercept	22.6% (n = 7)	-	
	Certolizumab	3.2% (n = 1)	-	
ESR (mm)		14 (6–31)^a^	-	
CRP (mg/L)		7.9 (3.0–23.2)^a^	-	
DAS28		4.24 (3.70–5.32)^a^	-	
DAS28-CRP		4.42 (3.69–5.11)^a^	-	
HAQ		1.63 (1–2)^a^	-	

^a^Results expressed as median and 25th and 75th percentiles. The Mann–Whitney test was used for quantitative variables; the Fisher exact test was used for other variables (male/female, tobacco precursor). *RA* rheumatoid arthritis, *BMI* body mass index, *RF* rheumatoid factor, *ACPA* anti-citrullinated peptide antibodies, *ESR* erythrocyte sedimentation rate, *CRP* C-reactive protein, *DAS28* Disease Activity Score in 28 joints, *HAQ* Health Assessment Questionnaire, *NSAID* nonsteroidal anti-inflammatory drugs

### Baseline lymphocyte subpopulations levels in patients and controls

We compared the frequencies of lymphocyte subpopulations in patients before the use of biologic drugs with those in controls (Table 2). The only statistically significant differences were in T2/Breg cells, which were lower in patients, both in frequency (%, *p* < 0.01) and in absolute numbers (*p* < 0.05), and Th17 frequency (*p* < 0.05).

**Table 2:** Initial lymphocyte subpopulations levels in patients with RA and controls

Lymphocyte populations	Patient group (n = 31)	Control group (n = 17)	*p*
Lymphocytes			
(%)	23.8 (17.0–30.0)	18.6 (13.6–28.5)	0.413
(/mm3)	1600 (1300–2000)	1700 (1050–1950)	0.974
B lymphocytes			
(%)	11.1 (8.2–17.6)	10.0 (7.3–14.4)	0.651
(/mm3)	156 (95–278)	151 (122–195)	0.957
TCD4+			
(%)	53.5 (47.0–60.9)	51.7 (44.8–58.1)	0.779
(/mm3)	723 (569–1081)	739 (549–955)	0.821
T2/Breg			
(%)	8.5 (5.9–10.5)	13.8 (10.9–15.6)	<0.001**
(/mm3)	13 (9–22)	17 (14–31)	0.014*
CD24hiCD27^+^ Breg			
(%)	16.3 (13.1–28.6)	18.8 (11.9–24.5)	0.612
(/mm3)	33 (20–45)	23 (19–34)	0.185
Treg			
(%)	6.4 (4.9–8.1)	7.1 (6.3–8.3)	0.223
(/mm3)	49 (35–65)	56 (44–68)	0.371
Th17			
(%)	19.6 (13.2–22.7)	23.4 (18.6–28.4)	0.036*
(/mm)	77 (45–97)	76 (51–135)	0.539

Total lymphocytes, B lymphocytes, T2/B regulatory cells (Breg), CD24^hi^CD27^+^ Breg, T regulatory cells (Treg) and T helper 17 (Th17) observed in patients with rheumatoid arthritis (RA) and controls in absolute value (cells per mm^3^) and percentage; expressed in median and IQR; difference observed between the two groups as per the Mann–Whitney test. *p < 0.05. **p < 0.001

### Association between the immunologic and radiographic status of patients and their lymphocyte subpopulations at baseline

We compared the frequencies of lymphocyte subpopulations before the use of biologic drugs in patients with positive (n = 23) or negative (n = 8) RF, positive (n = 25) or negative (n = 6) ACPA and with or (n = 13) or without (n = 18) structural damage. There was an association between the presence of RF and lower absolute numbers of T2/Breg (*p* = 0.016) with a median of 10.99/mm^3^ (8.12–16.58) for RF^+^ patients vs. 23.1/mm^3^ (16.03–24.74) for RF^-^ patients. No further association was found between other lymphocyte subpopulations and ACPA or radiographic status.

### Correlation between lymphocyte subpopulation levels in patients and controls

We tested potential correlation between T2/Breg, Treg and Th17 frequencies or absolute values, in controls and patients at different time points during treatment. In controls (n = 17), there was a trend toward correlation between the absolute number of Treg and T2/Breg (*r* = 0.500, *p* = 0.041) and between Treg and Th17 cells (*r* = 0.740 and *p* = 0.001).

In patients at baseline (n = 31), the positive correlation between absolute numbers of Treg and Th17 cells was confirmed (*r* = 0.579, *p* = 0.001) and between CD24^hi^CD27^+^ B cells and Th17 cells (*r* = 0.375, *p* = 0.037). At M1, there was positive correlation between absolute numbers of Treg and Th17 cells (*r* = 0.466, *p* = 0.011) and at M6 (*r* = 0.497 and *p* = 0.010); between absolute values of T2/Breg and Th17 cells at M1 (*r* = 0.385 and *p* = 0.039) and at M3 in percentage values (*r* = 0.383 and *p* = 0.040) and positive correlation between absolute values of CD24^hi^CD27^+^ Breg and Th17 cells only at M1 (*r* = 0.513 and *p* = 0.004).

### Evolution of lymphocyte subpopulations levels under biologic drugs

We compared the evolution of the lymphocyte subpopulations in patients between M0 (n = 31), M1 (n = 29), M3 (n = 26) and M6 (n = 26). We observed a difference only in Treg percentage between the four measurement times (*p* = 0.048). No significant evolution between the different treatment duration was observed for percentage or for absolute numbers in any of the other subsets (Table 3).

**Table 3:** Evolution of lymphocyte subpopulations levels in patients with RA under biologic drugs

Lymphocyte populations	Patients M0 (n = 31)	Patients M1 (n = 29)	Patients M3 (n = 29)	Patients M6 (n = 26)	*p* (6 months)
Lymphocytes					
(%)	23.8 (18.2–29.9)	29.8 (17.9–36.7)	28.9 (22.9–33.4)	29.55 (20.95–36.8)	0.140
(/mm)	1600 (1300–2000)	1600 (1300–2200)	1900 (1500–2300)	1550 (1300–1900)	0.117
B lymphocytes					
(%)	11.07 (8.28–16.05)	11.60 (8.94–17.47)	12.42 (8.44–15.96)	10.365 (8.38–14.52)	0.077
(/mm3)	156.15 (95.5–270.2)	201.57 (143.02–314.80)	192.63 (122.65–260.56)	180.66 (119.86–255.99)	0.989
T2/Breg					
(%)	8.46 (5.95–10.35)	6.43 (4.31–9.92)	8.54 (6.35–12.5)	9.35 (5.93–12.65)	0.099
(/mm)	12.75 (8.88–21.15)	13.47 (8.85–24.43)	15.29 (9.06–28.36)	15.81 (8.40–22.83)	0.489
CD24^hi^CD27^+^ Breg					
(%)	16.34 (13.2–27.71)	18.53 (15.02–31.61)	20.16 (14.81–30.26)	23.33 (14.02–33.00)	0.246
(/mm3)	33.26 (20.1–43.84)	34.87 (23.29–65.00)	35.22 (22.38–66.29)	35.54 (17.28–55.98)	0.813
Treg					
(%)	6.37 (4.91–7.98)	5.41 (4.36–7.06)	5.54 (4.63–6.85)	6.38 (4.87–7.02)	0.048*
(/mm3)	49.40 (37.6–62.52)	40.36 (30.60–60.24)	47.7 (35.89–65.28)	51.20 (34.97–66.14)	0.702
Th17					
(%)	19.57 (13.4–22.48)	19.0 (12.87–24.39)	19.01 (14.63–23.90)	19.50 (14.40–25.85)	0.928
(/mm3)	76.85 (49.1–96.77)	76.4 (47.75–112.7)	77.10 (61.80–113.43)	77.27 (65.66–91.44)	0.958

Total lymphocytes, B lymphocytes, T2/B regulatory cells (Breg), CD24^hi^CD27^+^ Breg, T regulatory cells (Treg) and T helper 17 (Th17) cells observed in absolute value (cells/mm^3^) and percentage (%) at different stages of the treatment, i.e. M0 (n = 31), M1 (n = 29), M3 (n = 29) and M6 (n = 26) and the difference observed; expressed as median and IQR, Kruskall − Wallis test. *RA* rheumatoid arthritis. *p < 0.05

We then compared the evolution of the lymphocyte subpopulations according to the type of treatment received: (1) abatacept, an “anti-costimulation” treatment (n = 12) vs. “anti-cytokine” (n = 19) comprising anti-TNF (n = 13) and tocilizumab (n = 6) and then (2) anti-TNF drug (n = 13) vs. abatacept and tocilizumab (n = 18). We observed that Treg frequencies significantly decreased over time under abatacept (*p* < 0.001), from a M0 median of 6.37% (5.26–7.23) to 4.68% (4.32–5.70) at M6; while the frequencies of Treg increased under anti-TNF drugs between M0 and M6 (*p* = 0.005), from a median of 6.49 (4.77–8.49) to 7.13 (6.69–7.95).

### Baseline levels and evolution of lymphocyte subpopulations in patients according to the DAS28 remission at M6

**Fig. 2:**
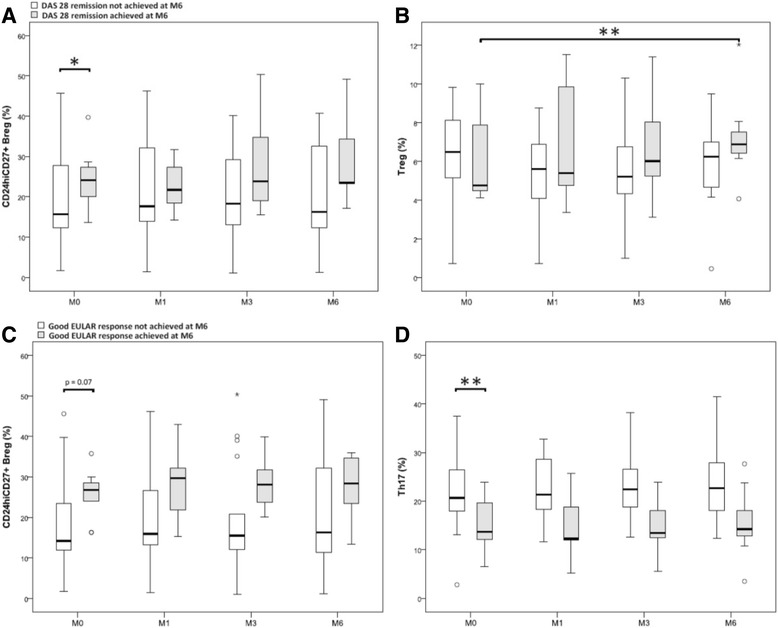
Baseline levels and evolution of lymphocyte subpopulations in patients with rheumatoid arthritis according to Disease Activity Score in 28 joints (*DAS28*) remission or European League Against Rheumatism (*EULAR*) response at month (M)6. **a** Baseline levels and evolution of CD24^hi^CD27^+^ B regulatory cells (*Breg*) in percentage between M0 and M6 in the patients who achieved DAS 28 remission (n = 7) or not (n = 19) at M6. **b** Initial levels and evolution of T regulatory cells (*Treg*) in percentage between M0 and M6 in the patients who achieved DAS28 remission (n = 7) or not (n = 19) at M6. **c** Initial levels and evolution of CD24^hi^CD27^+^ Breg in percentage between M0 and M6 in patients who achieved good EULAR response at M6 (n = 9) or not (n = 17). **d** Baseline levels and evolution of T helper 17 (Th17) cells in percentage between M0 and M6 in patients who achieved good EULAR response at M6 (n = 9) or not (n = 17). Results in box plots are median and IQR; difference observed between the two groups was assessed by the Mann–Whitney test at M0 and the general linear model (GLM) procedure and MIXED SAS for repetitive analyses. *p < 0.05, **p < 0.01

We attempted to determine whether the initial level of the different lymphocyte subpopulations studied was associated with DAS28 remission at M6. We dichotomised patients who achieved DAS28 remission (Fig. 2, dark grey box plot (n = 7)), or not (white box plot, (n = 19)) at M6. We then observed the evolution of these different populations at M1, M3 and M6. A higher baseline frequency of CD24^hi^CD27^+^ B cells was associated with DAS28 remission at M6 (*p* = 0.04) (Fig. 2a) with a median of 24.13% (20.04–27.34) for the patients in remission at M6 vs. 15.66% (12.31–27.72) for the others. The Treg increased during treatment in patients achieving DAS28 remission at M6 (p = 0.01), their median increasing from 4.76% (4.21–7.89) at M0 to 6.90% (6.43–7.22) at M6 (Fig. 2b).

### Baseline levels and evolution of lymphocyte subpopulations in patients according to the EULAR response at M6

We repeated this analysis with respect to achieving a good response (n = 9) or not (n = 17) using EULAR criteria at M6. A higher baseline frequency of CD24^hi^CD27^+^ B cells was associated with a good EULAR response at M6 but this was not statistically significant (*p* = 0.130), with a median of 26.79% (8.13–28.64) at M0 in patients with a good EULAR response at M6 vs. 14.22% (11.66–22) in the others.

A trend was observed in favour of a higher frequency of CD24^hi^CD27^+^ B cell in percentage at each of the treatment duration time points in patients with a good EULAR response at M6 vs. the others (*p* = 0.07) (Fig. 2c).

A lower frequency of Th17 cells before treatment was significantly associated with a good EULAR response at M6 (*p* = 0.007) with a median of 13.73% (12.1–19.57) at M0 in patients with a good EULAR response at M6 vs. 20.66% (17.92–26.16) in the others. An identical trend was observed in absolute numbers (*p* = 0.06) with a Th17 median of 13.73/mm^3^ (12.1–19.57) for patients with a good response at M6 vs. 20.66/mm^3^ (17.92–26.16) for the others. Likewise, a significantly lower frequency of Th17 cells was recorded at each treatment duration time point in patients with a good EULAR response at M6 vs. the others (*p* = 0.009), and the same trend was observed in absolute numbers (*p* = 0.06) (Fig. 2d).

The initial level of T2/Breg and Treg cells was not associated with good EULAR response at M6. Finally, there was no significant difference in the evolution of the other lymphocyte subpopulations under treatment, whether the patient did or not have a good EULAR response at M6.

### Baseline lymphocyte subpopulations levels according to type of biologic drugs used

We analysed out data again with respect to good EULAR response at M6, according to the type of biologic drug received, i.e. abatacept (n = 10), anti-cytokine (anti-TNF drugs and tocilizumab, (n = 16)) or anti-TNF drugs only (n = 10). The proportion of patients with a good EULAR response at M6 was 4/10 with abatacept, 2/6 with tocilizumab and 3/10 with anti-TNF drugs.

**Fig. 3:**
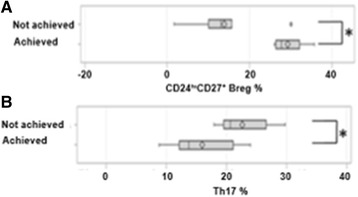
Baseline lymphocyte subpopulation levels according to the European League Against Rheumatism (*EULAR*) response at month 6 (*M6*) by type of biologic drug. **a** Comparison of baseline levels of CD24^hi^CD27^+^ B regulatory cells (*Breg*) in percentage, between the patients who achieved a good EULAR response at M6 (n = 4) or not (n = 6) under abatacept. **b** Comparison of T helper 17 cell (*Th17*) levels in percentage at M0 between patients who achieved good EULAR response at M6 (n = 5) or not (n = 11) under anti-cytokine treatment; box-plots show median and IQR; difference observed between the two groups using the *t* test. *p < 0.05

A high frequency of CD24^hi^CD27^+^ B cells was associated with a good EULAR response at M6 under abatacept (*p* = 0.01) (Fig. 3a), with a median of 26.79% (10.29–30.43) for patients with good response vs. 13% (10.32–15.19). The initial level of the other lymphocyte subpopulations was not associated with this response. A low initial level of Th17 cells was associated with a good EULAR response at M6 with the use of anti-cytokine (Fig. 3b, *p* = 0.048) with a median of 13.73% (12.1–21.13) in patients with good response vs. 20.62% (18.55–25.08) in the others. The initial level of the other lymphocyte subpopulations was not associated with this response.

## Discussion

Our data describe the effects of biologic drugs on B cells (CD24^hi^CD27^+^ Breg and T2/Breg), Treg and Th17 subsets, immuno-regulating lymphocyte subpopulations, involved in numerous autoimmune pathologic conditions such as RA. In our study, participants were representative of the patients commonly encountered in clinical practice (2/3 women, median age approximately 60 years). One third of the RA had developed within the previous 2 years. The median DAS28 was 4.24 and 3/4 of the subjects had immunopositive (RF and/or CCP+) RA and almost 50% of them had erosive RA.

In our study, T2/Breg cells were significantly reduced, both in percentage and in absolute numbers, in patients compared with controls. Other studies report a quantitative decrease in Breg in patients with RA compared to healthy control subjects and in patients with active RA compared with inactive disease [30, 31]. This lymphocyte subpopulation may therefore represent an objective biomarker of the activity of the disease. Moreover, we observed positive correlation in absolute numbers between T2/Breg and Treg cells in controls but not in patients, both at inclusion and during treatment, which may reflect dysfunction of the T2/Breg-Treg cell balance in RA. The T2/Bregs were also lower in patients with RF, as described in another study [30], in which negative correlation was observed between Bregs and RF and ACPA levels. There is no consensus as yet on the role of these antibodies in RA, but it has been proven that their presence is a marker of poor prognosis. Our data showing lower T2/Breg levels in patients with RA compared to controls, and in RF^+^ patients with RA, may therefore indicate a “protective” role.

DAS28 remission at M6 was associated with significantly higher CD24^hi^CD27^+^Breg frequency at baseline. Likewise, higher CD24^hi^CD27^+^ Breg frequency was initially observed in patients who had a good EULAR response at M6 (although not significant). The literature suggests that higher pre-treatment levels of CD24^hi^CD27^+^ Breg and T2/Breg may be predictive of a good EULAR response at 3 months with anti-TNF treatment [25]. A retrospective study of patients with RA receiving abatacept, showed that the level of CD24^hi^CD27^+^ Breg prior to treatment was not significantly different between patients who were EULAR responders and non-responder patients at M6 [34], whereas our results showed that a high initial level of CD24^hi^CD27^+^ Breg is significantly associated with a good EULAR response at M6 under abatacept. Therefore, CD24^hi^CD27^+^ Breg, that were higher at M0 in patients with DAS28 remission and in patients with good EULAR responses at M6, may represent a predictive biomarker for response to treatment, notably with abatacept. Finally, the activity of the disease (evaluated by DAS28) correlated negatively with the proportion of T2/Breg cells (and CD5^+^CD1d^hi^ [25] and IL-10 + CD5Cd1d^hi^ and IL-10^+^TIM-1^+^ [30, 31]) which also suggest a potential of this subset for disease monitoring.

Several studies reported more Treg in healthy subjects than in patients with early RA, but others reported similar levels [35–37]. Our data showed no significant difference in Treg at baseline between the patients and the controls, most likely due to the small number of participants in our current work, as we previously identified significant differences with larger numbers of subjects [38]. Whereas the frequency of Treg cells was similar at baseline in patients treated with the different drugs (anti-TNF, tocilizumab and abatacept), we observed a decrease in Tregs under abatacept but an increase under anti-TNF drugs. As previously described [6, 7], this may be partly explained by the removal of the repression exerted by this major proinflammatory cytokine on these cells.

In our study, the pre-treatment level of Treg was not predictive of DAS28 remission or EULAR response at M6. However, we observed a significant evolution of Treg under biologic drugs, with a recovery of these cells in patients with DAS28 remission at M6. Their level is described in the literature as being higher in drug-naive patients with early disease, who will achieve remission with MTX [38] and recovery of these cells under anti-TNF drugs was associated with a greater probability of long-term remission on anti-TNF drugs [7]. Other authors also report an increase in Treg over the course of anti-TNF [6] and abatacept [8] treatment only in patients with a good response. Treg may therefore represent an efficient biomarker for monitoring patients but not for predicting their personal response.

Th17 cell levels were significantly lower in patients than in the control group. Another study, however, described elevated levels of Th17 cells in RA, but only in patients with recent onset of disease [18]. There is a large amount of literature on such quantification with major discrepancies due to the use of different techniques (with or without a polarization-inducing condition) and the use of different phenotypes to identify Th17 cells [39, 40]. We did not find any association between the level of Th17 cells and the presence or absence of ACPA, although inverse correlation between the frequency of Th17 cells in the blood and the ACPA level had been previously described [17]. In addition, there was positive correlation between the absolute numbers of CD24^hi^CD27^+^ Breg and Th17 cells in patients with RA at baseline, but not in the controls. This was no longer observed after 3 and 6 months of treatment. This would therefore suggest initial pathologic correlation in RA, which disappeared over the course of a biology-modifying treatment such as a biologic drug.

Reduction in Th17 cells under MTX [18] and increase in non-response to infliximab [19] have been described In our study, patients with a good EULAR response at M6 had a significantly lower frequency of Th17 cells compared with moderate responders or non-responders, both at inclusion and at all the treatment points. A small proportion of Th17 cells at baseline was associated with a good EULAR response at M6 under anti-cytokine treatments but not under abatacept. The levels also did not evolve under treatment, whether the patients responded or not, which possibly reflects a lack of implication of this cell population in RA at late stages of the disease continuum (i.e. DMARD-resistant, biologic-drug-naive), which appears to contribute to the explanation of the disappointing results with treatments targeting IL-17 in clinical trials among patients with established RA [41]. In contrast, the positive correlation between the absolute numbers of Treg and Th17 cells, both in the controls and in the patient groups is likely to reinforce the hypothesis of an early role of Th17 cells in establishing chronicity [18]. It should finally be noted that low levels of Th17 cells may have predictive value for response to “anti-cytokine” treatments.

## Conclusions

This work aimed to establish pilot data using routine immunology hospital services protocols towards describing the evolution of different lymphocyte subpopulations in patients with RA under biologic drugs with different mechanisms of action, and assessing whether there is correlation between their levels and the later therapeutic response. Whereas, to date, only RF and ACPA are used to attempt to stratify patients for personalised treatment, CD24^hi^CD27^+^ B cells and Th17 cells may offer predictive value as biomarkers of therapeutic response under biologic drugs and may be able to guide clinicians in their choice of a tailored treatment. If validated, our data would suggest the prescribing of abatacept if the patient has high baseline levels of CD24^hi^CD27^+^ B cells, whereas a low initial level of Th17 cells may guide the choice of an anti-cytokine. As this was a pilot study, it remains crucial that our results are confirmed on a wider scale before being used to guide our therapeutic strategies.

## Abbreviations

ACPA: Anticitrullinated peptide antibody
ACR/EULAR: American College of Rheumatology/European League Against Rheumatism
BMI: Body mass index
Breg: Regulatory B lymphocytes
CMF: Flow cytometry
CRP: C-reactive protein
DAS28: Disease Activity Score in 28 joints
DMARD: Disease-modifying antirheumatic drug
ESR: Erythrocyte sedimentation rate
HAQ: Health Assessment Questionnaire
IL: Interleukin
MTX: Methotrexate
PBMC: Peripheral blood mononuclear cell
RA: Rheumatoid arthritis
RF: Rheumatoid factor
T2T: Treat to target
Th17: T helper type 17 cell
TNF: Tumour necrosis factor
Treg: regulatory T cells
